# Automatic detection of cyberbullying in social media text

**DOI:** 10.1371/journal.pone.0203794

**Published:** 2018-10-08

**Authors:** Cynthia Van Hee, Gilles Jacobs, Chris Emmery, Bart Desmet, Els Lefever, Ben Verhoeven, Guy De Pauw, Walter Daelemans, Véronique Hoste

**Affiliations:** 1 Department of Translation, Interpreting and Communication - Faculty of Arts and Philosophy, Ghent University, Ghent, Belgium; 2 Department of Linguistics - Faculty of Arts, University of Antwerp, Antwerp, Belgium; University of Cape Town, SOUTH AFRICA

## Abstract

While social media offer great communication opportunities, they also increase the vulnerability of young people to threatening situations online. Recent studies report that cyberbullying constitutes a growing problem among youngsters. Successful prevention depends on the adequate detection of potentially harmful messages and the information overload on the Web requires intelligent systems to identify potential risks automatically. The focus of this paper is on automatic cyberbullying detection in social media text by modelling posts written by bullies, victims, and bystanders of online bullying. We describe the collection and fine-grained annotation of a cyberbullying corpus for English and Dutch and perform a series of binary classification experiments to determine the feasibility of automatic cyberbullying detection. We make use of linear support vector machines exploiting a rich feature set and investigate which information sources contribute the most for the task. Experiments on a hold-out test set reveal promising results for the detection of cyberbullying-related posts. After optimisation of the hyperparameters, the classifier yields an F_1_ score of 64% and 61% for English and Dutch respectively, and considerably outperforms baseline systems.

## Introduction

Web 2.0 has had a substantial impact on communication and relationships in today’s society. Children and teenagers go online more frequently, at younger ages, and in more diverse ways (e.g. smartphones, laptops and tablets). Although most of teenagers’ Internet use is harmless and the benefits of digital communication are evident, the freedom and anonymity experienced online makes young people vulnerable with cyberbullying being one of the major threats [1, 2].

Bullying is not a new phenomenon and cyberbullying has manifested itself as soon as digital technologies have become primary communication tools. On the positive side, social media like blogs, social networking sites (e.g. Facebook), and instant messaging platforms (e.g. WhatsApp) make it possible to communicate with anyone and at any time. Moreover, they are a place where people engage in social interaction, offering the possibility to establish new relationships and maintain existing friendships [3, 4]. On the negative side however, social media increase the risk of children being confronted with threatening situations including grooming or sexually transgressive behaviour, signals of depression and suicidal thoughts, and cyberbullying. Users are reachable 24/7 and are often able to remain anonymous if desired: this makes social media a convenient way for bullies to target their victims outside the school yard.

With regard to cyberbullying, a number of national and international initiatives have been launched over the past few years to increase children’s online safety. Examples include *KiVa* (http://www.kivaprogram.net/), a Finnish cyberbullying prevention programme, the ‘*Non au harcèlement*’ campaign in France, Belgian governmental initiatives and helplines (e.g. *clicksafe.be, veiligonline.be, mediawijs.be*) that provide information about online safety, and so on.

In spite of these efforts, a lot of undesirable and hurtful content remains online. [2] analysed a body of quantitative research on cyberbullying and observed cybervictimisation rates among teenagers between 20% and 40%. [5] focused on 12 to 17 year olds living in the United States and found that no less than 72% of them had encountered cyberbullying at least once within the year preceding the questionnaire. [6] surveyed 9 to 26 year olds in the United States, Canada, the United Kingdom and Australia, and found that 29% of the respondents had ever been victimised online. A study among 2,000 Flemish secondary school students (age 12 to 18) revealed that 11% of them had been bullied online at least once in the six months preceding the survey [7]. Finally, the 2014 large-scale EU Kids Online Report [8] published that 20% of 11 to 16 year olds had been exposed to hate messages online. In addition, youngsters were 12% more likely to be exposed to cyberbullying as compared to 2010, which clearly demonstrates that cyberbullying is a growing problem.

The prevalence of cybervictimisation depends on the conceptualisation used in describing cyberbullying, but also on research variables such as location and the number and age span of the participants. Nevertheless, the above studies demonstrate that online platforms are increasingly used for bullying, which is a cause for concern given its impact. As shown by [9–11], cyberbullying may negatively impact the victim’s self-esteem, academic achievement and emotional well-being. [12] found that self-reported effects of cyberbullying include negative effects on school grades and feelings of sadness, anger, fear, and depression. In extreme cases, cyberbullying could even lead to self-harm and suicidal thoughts.

These findings demonstrate that cyberbullying is a serious problem the consequences of which can be dramatic. Early detection of cyberbullying attempts is therefore of key importance to youngsters’ mental well-being. Successful detection depends on effective monitoring of online content, but the amount of information on the Web makes it practically unfeasible for moderators to monitor all user-generated content manually. To tackle this problem, intelligent systems are required that process this information in a fast way and automatically signal potential threats. This way, moderators can respond quickly and prevent threatening situations from escalating. According to recent research, teenagers are generally in favour of such automatic monitoring, provided that effective follow-up strategies are formulated, and that privacy and autonomy are guaranteed [13].

Parental control tools (e.g. *NetNanny*, https://www.netnanny.com/) already block unsuited or undesirable content and some social networks make use of keyword-based moderation tools (i.e. using lists of profane and insulting words to flag harmful content). However, such approaches typically fail to detect implicit and subtle forms of cyberbullying in which no explicit vocabulary is used. This creates the need for intelligent and self-learning systems that go beyond keyword spotting and hence improve the recall of cyberbullying detection.

The ultimate goal of this type of research is to develop models that could improve manual monitoring for cyberbullying on social networks. We explore the automatic detection of textual signals of cyberbullying, in which cyberbulying is approached as a complex phenomenon that can be realised in various ways (see the Annotation guidelines section for a detailed overview). While the vast majority of the related research focuses on detecting cyberbullying ‘attacks’ (i.e. verbal aggression), the present study takes different types of cyberbullying into account, including more implicit posts from the bully, but also posts written by victims and bystanders. This is a more inclusive conceptualisation for the task of cyberbullying detection and should aid in moderation and prevention efforts by capturing different and more implicit signals of bullying.

To tackle this problem, we propose a machine learning method based on a linear SVM classifier [14, 15] exploiting a rich feature set. The contribution we make is twofold: first, we develop a complex classifier to detect *signals* of cyberbullying, which allows us to detect different types of cyberbullying that are related to different social roles involved in a cyberbullying event. Second, we demonstrate that the methodology is easily portable to other languages, provided there is annotated data available, by performing experiments on an English and Dutch dataset.

The remainder of this paper is structured as follows: the next section presents a definition of cyberbullying and its participant roles and provides an overview of the state of the art in cyberbullying detection. The *Data collection and annotation* section describes the corpus construction and annotation. Next, we present the experimental setup and discuss our experimental results for English and Dutch. Finally, the *Conclusion and future research* section concludes this paper and provides some perspectives for further research.

## Related research

Both offline and online bullying are widely covered in the realm of social sciences and psychology, and the increasing number of cyberbullying cases in recent years [16] has stimulated research efforts to detect cyberbullying automatically. In the following section, we present a definition of cyberbullying and identify its participant roles and we provide a brief overview of automatic approaches to cyberbullying detection.

### Cyberbullying definition and participant roles

A common starting point for conceptualising cyberbullying are definitions of traditional (i.e. *offline*) bullying, one of the most influential ones being formulated by [17]. The researcher described bullying based on three main criteria, including i) **intention** (i.e. a bully intends to inflict harm on the victim), ii) **repetition** (i.e. bullying acts take place repeatedly over time) and iii) a **power imbalance** between the bully and the victim (i.e. a more powerful bully attacks a less powerful victim). With respect to cyberbullying, a number of definitions are based on the above criteria. A popular definition is that of [18, p. 376], which describes cyberbullying as “an aggressive, intentional act carried out by a group or individual, using electronic forms of contact, repeatedly and over time, against a victim who cannot easily defend him or herself”. However, opinion on the applicability of the above characteristics to cyberbullying is very much divided [19], and besides theoretical objections, a number of practical limitations have been observed. Firstly, while [17] claims intention to be inherent to traditional bullying, this is much harder to ascertain in an online environment. Online conversations lack the signals of a face-to-face interaction like intonation, facial expressions and gestures, which makes them more ambiguous than real-life conversations. The receiver may therefore get the wrong impression that they are being offended or ridiculed [20]. Another criterion for bullying that might not hold in online situations is the power imbalance between the bully and the victim. This can be evident in real life (e.g. the bully is taller, stronger or older than the victim), but it is hard to conceptualise or measure online, where power may be related to technological skills, anonymity or the inability of the victim to escape from the bullying [19, 21]. Also empowering for the bully are inherent characteristics of the Web: once defamatory or confidential information is made public through the Internet, it is hard to remove.

Finally, while arguing that repetition distinguishes bullying from single acts of aggression, [17] himself states that such a single aggressive action can be considered bullying under certain circumstances. Accordingly, [21] claim that repetition in cyberbullying is problematic to operationalise, as it is unclear what the consequences are of a single derogatory message on a public page. A single act of aggression or humiliation may cause continued distress and humiliation for the victim if it is shared or liked by a large audience [21]. [22, p. 26] compare this with the “snowball effect”: one post may be repeated or distributed by other people so that it becomes out of the control of the initial bully and has larger effects than was originally intended.

Given these arguments, a number of less ‘strict’ definitions of cyberbullying were proposed by among others [2, 5, 6], where a power imbalance and repetition are not deemed necessary conditions for cyberbullying.

The above paragraphs demonstrate that defining cyberbullying is far from trivial, and varying prevalence rates (see the Introduction section) confirm that a univocal definition of the phenomenon is still lacking in the literature [2]. Based on existing conceptualisations, we define cyberbullying as *content that is published online by an individual and that is aggressive or hurtful against a victim*. Based on this definition, an annotation scheme was developed [23] to signal textual characteristics of cyberbullying, including posts from bullies, as well as reactions from victims and bystanders.

Cyberbullying research also involves the identification of its participant roles. [24] were among the first to define the roles in a bullying situation. Based on surveys among teenagers involved in real-life bullying situations, they defined six participant roles: victims (i.e. who are the target of repeated harassment), bullies (i.e. who are the initiative-taking perpetrators), assistants of the bully (i.e. who encourage the bullying), reinforcers of the bully (i.e. who reinforce the bullying), defenders (i.e. who comfort the victim, take their side or try to stop the bullying) and outsiders (i.e. who ignore or distance themselves from the situation). In sum, in addition to the bully and victim, the researchers distinguish four bystanders (i.e. assistants, reinforcers, defenders and outsiders). [25], however, do not distinguish between reinforcers and assistants of the bullying. Their typology includes victims, bullies and three types of bystanders: i) bystanders who participate in the bullying, ii) bystanders who help or support the victim and iii) bystanders who ignore the bullying. The cyberbullying roles that are identified in our annotation scheme are based on existing bullying role typologies, given that traditional bullying roles are applicable to cyberbullying as well [26, 27]. More details about the different roles that we take into account are provided in the Data collection and annotation section.

Bystanders and -to a lesser extent- victims are often overlooked in the related research. As a result, these studies can be better characterised as verbal aggression detection concerned with retrieving bully attacks. By taking bystanders into account, we capture different and more subtle signals of a bullying episode. Note that while in this work we did not include classification of the participant roles as such, they are essential to the conceptualisation of the current detection task.

### Detecting and preventing cyberbullying

As mentioned earlier, although research on cyberbullying detection is more limited than social studies on the phenomenon, some important advances have been made in recent years. In what follows, we present a brief overview of the most important natural language processing approaches to cyberbullying detection, but we refer to the survey paper by [28] for a more detailed overview.

Although some studies have investigated the effectiveness of rule-based modelling [29], the dominant approach to cyberbullying detection involves machine learning. Most machine learning approaches are based on supervised [30, 30–32] or semi-supervised learning [33]. The former involves the construction of a classifier based on labelled training data, whereas semi-supervised approaches rely on classifiers that are built from a training corpus containing a small set of labelled and a large set of unlabelled instances. Semi-supervised methods are often used to handle data sparsity, a typical issue in cyberbullying research. As cyberbullying detection essentially involves the distinction between bullying and non-bullying posts, the problem is generally approached as a binary classification task where the positive class is represented by instances containing (textual) cyberbullying, while the negative class is devoid of bullying signals.

A key challenge in cyberbullying research is the availability of suitable data, which is necessary to develop models that characterise cyberbullying. In recent years, only a few datasets have become publicly available for this particular task, such as the training sets provided in the context of the CAW 2.0 workshop (http://caw2.barcelonamedia.org), a MySpace (https://myspace.com) [34] and Formspring (http://www.formspring.me) cyberbullying corpus annotated with the help of Mechanical Turk [29], and more recently, the Twitter Bullying Traces dataset [35]. Many studies have therefore constructed their own corpus from social media websites that are prone to bullying content, such as YouTube [30, 32], Twitter [36, 37], Instagram [38], MySpace [31, 34], FormSpring [29, 39], Kaggle [40] and ASKfm [41]. Despite the bottleneck of data availability, cyberbullying detection approaches have been successfully implemented over the past years and the relevance of automatic text analysis techniques to ensure child safety online has been recognised [42].

Among the first studies on cyberbullying detection are [29–31], who explored the predictive power of *n*-grams (with and without tf-idf weighting), part-of-speech information (e.g. first and second pronouns), and sentiment information based on (polarity and profanity) lexicons for this task. Similar features were not only exploited for coarse-grained cyberbullying detection, but also for the detection of more fine-grained cyberbullying categories [41]. Despite their apparent simplicity, content-based features (i.e. lexical, syntactic and sentiment information) are very often exploited in recent approaches to cyberbullying detection [33, 43]. In fact, as observed by [28], more than 41 papers have approached cyberbullying detection using content-based features, which confirms that this type of information is crucial for the task.

More and more, however, content-based features are combined with semantic features derived from topic model information [44], word embeddings and representation learning [43, 45]. More recent studies have also demonstrated the added value of user-based information for the task, more specifically by including users’ activities (i.e. the number of posts) on a social network, their age, gender, location, number of friends and followers, and so on [32, 33, 46, 47]. A final feature type that gains increasing popularity in cyberbullying detection are network-based features, whose application is motivated by the frequent use of social media data for the task. By using network information, researchers aim to capture social relations between participants in a conversation (e.g. bully versus victim), and other relevant information such as the popularity of a person (i.e. which can indicate the power of a potential bully) on a social network, the number of (historical) interactions between two people, and so on. [48] for instance used network-based features to take the behavioural history of a potential bully into account. [49] detected cyberbullying in tweets and included network features inspired by Olweus’ [17] bullying conditions (see supra). More specifically, they measured the power imbalance between a bully and victim, as well as the bully’s popularity based on interaction graphs and the bully’s position in the network.

As mentioned earlier, social media are a commonly used genre for this type of tasks. More recently, researchers have investigated cyberbullying detection in multi-modal data offered by specific platforms. For instance [38] explored cyberbullying detection using multi-modal data extracted from the social network Instagram. More precisely, they combined textual features derived from the posts themselves with user metadata and image features and showed that integrating the latter enhanced the classification performance. [37] also detected cyberbullying in different data genres, including ASKfm, Twitter, and Instagram. They took role information into account by integrating bully and victim scores as features, based on the occurrence of bully-related keywords in their sent or received posts.

With respect to the datasets used in cyberbullying research, it can be observed that corpora are often composed by keyword search (e.g. [43, 44]), which produces a biased dataset of positive (i.e. bullying) instances. To balance these corpora, negative data are often added from a background corpus or data resampling [50] techniques are adopted [33, 47]. For this research, data were randomly crawled across ASKfm and no keyword search was used to collect bullying data. Instead, all instances were manually annotated for the presence of bullying. As a result, our corpus contains a realistic distribution of bullying instances.

When looking at the performance of automatic cyberbullying, we see that scores vary greatly and do not only depend on the implemented algorithm and parameter settings, but also on a number of other variables. These include the metrics that are used to evaluate the system (i.e. micro- or macro-averaged F_1_, precision, recall, AUC, etc.), the corpus genre (i.e. Facebook, Twitter, ASKfm, Instagram) and class distribution (i.e. balanced or unbalanced), the annotation method (i.e. automatic annotations or manual annotations using crowdsourcing or by experts) and, perhaps the most important distinguishing factor, the conceptualisation of cyberbullying that is used. More concretely, while some approaches identify sensitive topics [30] or insulting language [29], others propose a more comprehensive approach by capturing different types of cyberbullying [41] or by modelling the bully-victim communications involved in a cyberbullying incident [37].

The studies discussed in this section demonstrated the variety of approaches that have been used to tackle cyberbullying detection. However, most of them focused on cyberbullying ‘attacks’, or posts written by a bully. Moreover, it is not entirely clear if different forms of cyberbullying were taken into account (e.g. sexual intimidation or harassment, or psychological threats), in addition to derogatory language or insults. In the present study, cyberbullying is considered a complex phenomenon comprising different forms of harmful online behaviour, which are described in more detail in our annotation scheme [23]. Purposing to facilitate manual monitoring efforts on social networks, we developed a system that automatically detects signals of cyberbullying, including attacks from bullies, as well as victim and bystander reactions, the latter of which are generally overlooked in related research.

Most similar to this research is the work by [44], [43, 45], who investigated bullying traces posted by different author roles (e.g. bully, victim, bystander, assistant, defender, reporter, accuser, reinforcer). However, they collected tweets using the keywords *bully, bullied* and *bullying*. As a result, their corpus contained many reports or testimonials of cyberbullying (example 1), instead of actual cyberbullying. Moreover, their method implies that cyberbullying signals that are devoid of such keywords are not included in the training corpus.

1 *“Some tweens got violent on the n train, the one boy got off after blows 2 the chest… Saw him cryin as he walkd away: (bullying not cool”* [44, *p. 658*]

What clearly distinguishes these works from the present is that their conceptualisation of cyberbullying is not explained. It is, in other words, not clear which type of posts are considered bullying and which are not. In the present research, we identify different types of bullying and all are included in the positive class of our experimental corpus.

For this research, English and Dutch social media data were annotated for fine-grained forms of cyberbullying, based on the actors involved in a cyberbullying incident. After preliminary experiments for Dutch [41, 51], we currently present an optimised cyberbullying detection method for English and Dutch and hereby show that the proposed methodology can easily be applied to different languages, provided that annotated data are available.

## Data collection and annotation

To be able to build representative models for cyberbullying, a suitable dataset is required. This section describes the construction of two corpora, English and Dutch, containing social media posts that are manually annotated for cyberbullying according to our fine-grained annotation scheme. This allows us to cover different forms and participants (or *roles*) involved in a cyberbullying event.

### Data collection

Two corpora were constructed by collecting data from the social networking site ASKfm, where users can create profiles and ask or answer questions, with the option of doing so anonymously. ASKfm data typically consists of question-answer pairs published on a user’s profile. The data were retrieved by crawling a number of seed profiles using the GNU Wget software (http://www.gnu.org/software/wget/) in April and October, 2013. After language filtering (i.e. non-English or non-Dutch content was removed), the experimental corpora comprised 113,698 and 78,387 posts for English and Dutch, respectively.

### Data annotation

Cyberbullying has been a widely covered research topic recently and studies have shed light on direct and indirect types of cyberbullying, implicit and explicit forms, verbal and non-verbal cyberbullying, and so on. This is important from a sociolinguistic point of view, but knowing what cyberbullying involves is also crucial to build models for automatic cyberbullying detection. In the following paragraphs, we present our data annotation guidelines [23] and focus on different types and roles related to the phenomenon.

### Types of cyberbullying

Cyberbullying research is mainly centered around the conceptualisation, occurrence and prevention of the phenomenon [1, 52, 53]. Sociolinguistic studies have identified different types of cyberbullying [12, 54, 55] and compared these types with forms of traditional or offline bullying [20]. Like traditional bullying, direct and indirect forms of cyberbullying have been identified. Direct cyberbullying refers to actions in which the victim is directly involved (e.g. sending a virus-infected file, excluding someone from an online group, insulting and threatening), whereas indirect cyberbullying can take place without awareness of the victim (e.g. *outing* or publishing confidential information, spreading gossip, creating a hate page on social networking sites) [20].

The present annotation scheme describes some specific textual categories related to cyberbullying, including threats, insults, defensive statements from a victim, encouragements to the harasser, etc. (see the Data collection and annotation section for a complete overview). All of these forms were inspired by social studies on cyberbullying [7, 20] and manual inspection of cyberbullying examples.

### Roles in cyberbullying

Similarly to traditional bullying, cyberbullying involves a number of participants that adopt well-defined roles. Researchers have identified several roles in (cyber)bullying interactions. Although traditional studies on bullying have mainly concentrated on bullies and victims [24], the importance of bystanders in a bullying episode has been acknowledged [56, 57]. Bystanders can support the victim and mitigate the negative effects caused by the bullying [57], especially on social networking sites, where they hold higher intentions to help the victim than in real life conversations [58]. [25] distinguish three main types of bystanders: i) bystanders who participate in the bullying, ii) who help or support the victim and iii) those who ignore the bullying. Given that passive bystanders are hard to recognise in online text, only the former two are included in our annotation scheme.

### Annotation guidelines

To operationalise the task of automatic cyberbullying detection, we elaborated a detailed annotation scheme for cyberbullying that is strongly embedded in the literature and applied it to our corpora. The applicability of the scheme was iteratively tested. Our final guidelines for the fine-grained annotation of cyberbullying are described in a technical report [23]. The objective of the scheme was to indicate several types of textual cyberbullying and verbal aggression, their severity, and the author participant roles. The scheme is formulated to be generic and is not limited to a specific social media platform. All messages were annotated in context (i.e. presented within their original content or conversation event) when available.

Essentially, the annotation scheme describes two levels of annotation. Firstly, the annotators were asked to indicate, at the message or post level, whether the text under investigation was related to cyberbullying. If the message was considered harmful and thus contained indications of cyberbullying, annotators identified the author’s participant role. Based on the literature on role-allocation in cyberbullying episodes [25, 59], four roles are distinguished in the annotation scheme, including victim, bully, and two types of bystanders.

1. **Harasser or bully:** person who initiates the bullying.
2. **Victim:** person who is harassed.
3. **Bystander-defender:** person who helps the victim and discourages the harasser from continuing his actions.
4. **Bystander-assistant:** person who does not initiate, but helps or encourages the harasser.

Secondly, at the sub-sentence level, the annotators were tasked with the identification of fine-grained text categories related to cyberbullying. In the literature, different forms of cyberbullying are identified [12, 54, 55] and compared with traditional bullying [20]. Based on these forms, the annotation scheme describes a number of textual categories that are often inherent to a cyberbullying event, such as threats, insults, defensive statements from a victim, encouragements to the harasser, etc. Most of the categories are related to direct forms of cyberbullying (as defined by [25]), while one is related to *outing* [25], an indirect form of cyberbullying, namely *defamation*. Additionally, a number of subcategories were defined to make the annotation scheme as concrete and distinctive as possible (e.g., *discrimination* as a subcategory of *insult*). All cyberbullying-related categories in the scheme are listed below, and an example post for each category is presented in Table 1.

- **Threat/blackmail:** expressions containing physical or psychological threats or indications of blackmail.
- **Insult:** expressions meant to hurt or offend the victim.
  - **General insult:** general expressions containing abusive, degrading or offensive language that are meant to insult the addressee.
  - **Attacking relatives:** insulting expressions towards relatives or friends of the victim.
  - **Discrimination:** expressions of unjust or prejudicial treatment of the victim. Two types of discrimination are distinguished (i.e. sexism and racism). Other forms of discrimination should be categorised as general insults.
- **Curse/exclusion:** expressions of a wish that some form of adversity or misfortune will befall the victim and expressions that exclude the victim from a conversation or a social group.
- **Defamation:** expressions that reveal confident or defamatory information about the victim to a large public.
- **Sexual Talk:** expressions with a sexual meaning or connotation. A distinction is made between innocent sexual talk and sexual harassment.
- **Defense:** expressions in support of the victim, expressed by the victim himself or by a bystander.
  - **Bystander defense:** expressions by which a bystander shows support for the victim or discourages the harasser from continuing his actions.
  - **Victim defense:** assertive or powerless reactions from the victim.
- **Encouragement to the harasser:** expressions in support of the harasser.
- **Other:** expressions that contain any other form of cyberbullying-related behaviour than the ones described here.

**Table 1:** Definitions and brat annotation examples of more fine-grained text categories related to cyberbullying.

Annotation category	Annotation example
Threat/blackmail	[I am going to find out who you are & I swear you are going to regret it.]^threat^
Insult	[Kill yourself] ^curs^ [you fucking mc slut!!!!]^gen. insult^ [NO ONE LIKES YOU!!!!!]^gen. insult^ [You are an ugly useless little whore!!!!] ^gen. insult^
Curse/Exclusion	[Fuck you.]^gen. insult^ [Now shush I don’t wanna hear anything.]^curse or exclusion^
Defamation	[She slept with her ex behind his girlfiends back and she and him had broken up.] ^defamation^
Sexual Talk	[Naked pic of you now.]^sexual harassment^
Defense	[I would appreciate if you dindn’t talk shit about my bestfriend.]^gen. victim defense^ He has enough to deal with already.
Encour. to har.	[She is a massive slut]^gen. insult^ [i agree with you @user she is!]^encour. harasser^ [LOL AT HER mate, im on your side]^encour. harasser^

It is important to note that the categories were always indicated in text, even if the post in which they occurred was not considered harmful, for instance in the post “hi bitches, in for a movie?”, “bitches” was annotated as an insult while the post itself was not considered cyberbullying.

To provide the annotators with some context, all posts were presented within their original conversation when possible. All annotations were done using the brat rapid annotation tool [60], some examples of which are presented in Table 1.

As can be deduced from the examples in the table, there were no restrictions as to what form the annotations could take. They could be adjectives, noun phrases, verb phrases, and so on. The only condition was that the annotation could not span more than one sentence and less than one word. Posts that were (primarily) written in another language than the corpus language (i.e. Dutch and English) were marked as such and required no further annotations.

We examined the validity of our guidelines and the annotations with an inter-annotator agreement experiment that is described in the following section.

### Annotation statistics

The English and Dutch corpora were independently annotated for cyberbullying by trained linguists. All were Dutch native speakers and English second-language speakers. To demonstrate the validity of our guidelines, inter-annotator agreement scores were calculated using Kappa on a subset of each corpus. Inter-rater agreement for Dutch (2 raters) is calculated using Cohen’s Kappa [61]. Fleiss’ Kappa [62] is used for the English corpus (> 2 raters). Kappa scores for the identification of cyberbullying are *κ* = 0.69 (Dutch) and *κ* = 0.59 (English).

As shown in Table 2, inter-annotator agreement for the identification of the more fine-grained categories for English varies from fair to substantial [63], except for *defamation*, which appears to be more difficult to recognise. No encouragements to the harasser were present in this subset of the corpus. For Dutch, the inter-annotator agreement is fair to substantial, except for *curse* and *defamation*. Analysis revealed that one of both annotators often annotated the latter as an insult, and in some cases even did not consider it as cyberbullying-related.

**Table 2:** Inter-annotator agreement on the fine-grained categories related to cyberbullying.

	Threat	Insult	Defense	Sexual talk	Curse/exclusion	Defamation	Encouragements to the harasser
**English**	0.65	0.63	0.45	0.38	0.58	0.15	N/A
**Dutch**	0.52	0.66	0.63	0.53	0.19	0.00	0.21

In short, the inter-rater reliability study shows that the annotation of cyberbullying is not trivial and that more fine-grained categories like *defamation*, *curse* and *encouragements* are sometimes hard to recognise. It appears that defamations were sometimes hard to distinguish from insults, whereas curses and exclusions were sometimes considered insults or threats. The analysis further reveals that encouragements to the harasser are subject to interpretation. Some are straightforward (e.g. “I agree we should send her hate”), whereas others are subject to the annotator’s judgment and interpretation (e.g. “hahaha”, “LOL”).

## Experimental setup

In this paper, we explore the feasibility of automatically recognising signals of cyberbullying. A crucial difference with related research is that we do not only model bully ‘attacks’, but also more implicit forms of cyberbullying and reactions from victims and bystanders (i.e. all under one binary label ‘signals of cyberbullying’), since these could likewise indicate that cyberbullying is going on. The experiments described in this paper focus on the automatic detection of such cyberbullying signals that need to be further investigated by human moderators when applied in a real-life moderation loop.

The English and Dutch corpus contain 113,698 and 78,387 posts, respectively. As shown in Table 3, the experimental corpus features a heavily **imbalanced** class distribution with the large majority of posts not being part of cyberbullying. In classification, this class imbalance can lead to decreased performance. We apply cost-sensitive SVM as a possible hyperparameter in optimisation to counter this. The cost-sensitive SVM reweighs the penalty parameter *C* of the error term by the inverse class-ratio. This means that misclassifications of the minority positive class are penalised more than classification errors on the majority negative class. Other pre-processing methods to handle data imbalance in classification include feature filtering metrics and data resampling [64]. These methods were omitted as they were found to be too computationally expensive given our high-dimensional dataset.

**Table 3:** Statistics of the English and Dutch cyberbullying corpus.

	Corpus size	Number(ratio) of bullying posts
**English**	113,698	5,375(4.73%)
**Dutch**	78,387	5,106(6.97%)

For the automatic detection of cyberbullying, we performed **binary classification experiments** using a linear kernel support vector machine (SVM) implemented in LIBLINEAR [65] by making use of Scikit-learn [66], a machine learning library for Python. The motivation behind this is twofold: i) support vector machines (SVMs) have proven to work well for tasks similar to the ones under investigation [67] and ii) LIBLINEAR allows fast training of large-scale data which allow for a linear mapping (which was confirmed after a series of preliminary experiments using LIBSVM with linear, RBF and polynomial kernels).

The classifier was optimised for feature type (see the Pre-processing and feature engineering section) and hyperparameter combinations (see Table 4). Model selection was done using 10-fold cross validation in grid search over all possible feature types (i.e. groups of similar features, like different orders of *n*-gram bag-of-words features) and hyperparameter configurations. The best performing hyperparameters are selected by F_1_ score on the positive class. The winning model is then retrained on all held-in data and subsequently tested on a hold-out test set to assess whether the classifier is over- or under-fitting. The hold-out set represents a random sample (10%) of all data. The folds were randomly stratified splits over the hold-in class distribution. Testing all feature type combinations is a rudimentary form of feature selection and provides insight into which types of features work best for this particular task.

**Table 4:** Hyperparameters in grid-search model selection.

Hyperparameter	Values
Penalty of error term *C*	1*e*^{−3, −2, …, 2,3}^
Loss function	Hinge, squared hinge
Penalty: norm used in penalisation	‘l1’ (‘least absolute deviations’) or ‘l2’ (‘least squares’)
Class weight (sets penalty *C* of class *i* to weight*C)	None or ‘balanced’, i.e. weight inversely proportional to class frequencies

Feature selection over all individual features was not performed because of the large feature space (NL: 795,072 and EN: 871,296 individual features). [68], among other researchers, demonstrated the importance of joint optimisation, where feature selection and hyperparameter optimisation are performed simultaneously, since the techniques mutually influence each other.

The optimised models are evaluated against **two baseline systems**: i) an unoptimised linear-kernel SVM (configured with default parameter settings) based on word *n*-grams only and, ii) a keyword-based system that marks posts as positive for cyberbullying if they contain a word from existing vocabulary lists composed by aggressive language and profanity terms.

### Pre-processing and feature engineering

As pre-processing, we applied tokenisation, PoS-tagging and lemmatisation to the data using the LeTs Preprocess Toolkit [69]. In supervised learning, a machine learning algorithm takes a set of training instances (of which the label is known) and seeks to build a model that generates a desired prediction for an unseen instance. To enable the model construction, all instances are represented as a vector of features (i.e. inherent characteristics of the data) that contain information that is potentially useful to distinguish cyberbullying from non-cyberbullying content.

We experimentally tested whether cyberbullying events can be recognised automatically by lexical markers in a post. To this end, all posts were represented by a number of information sources (or *features*) including lexical features like bags-of-words, sentiment lexicon features and topic model features, which are described in more detail below. Prior to feature extraction, some data cleaning steps were executed, such as the replacement of hyperlinks and @-replies, removal of superfluous white spaces, and the replacement of abbreviations by their full form (based on an existing mapping dictionary: http://www.chatslang.com/terms/abbreviations/). Additionally, tokenisation was applied before *n*-gram extraction and sentiment lexicon matching, and stemming was applied prior to extracting topic model features.

After pre-processing of the corpus, the following feature types were extracted:

- **Word *n*-gram bag-of-words:** binary features indicating the presence of word unigrams, bigrams and trigrams.
- **Character *n*-gram bag-of-words:** binary features indicating the presence of character bigrams, trigrams and fourgrams (without crossing word boundaries). Character *n*-grams provide some abstraction from the word level and provide robustness to the spelling variation that characterises social media data.
- **Term lists:** one binary feature derived for each one out of six lists, indicating the presence of an item from the list in a post:
  - proper names: a gazetteer of named entities collected from several resources (e.g. Wikipedia).
  - ‘allness’ indicators (e.g. “always”, “everybody”): forms which indicate rhetorical superlativity [70] which can be helpful in identifying the often hyperbolic bullying language.
  - diminishers (e.g. “slightly”, “relatively”): diminishers, intensifiers and negation words were all obtained from an English grammar describing these lexical classes and existing sentiment lexicons (see further).
  - intensifiers (e.g. “absolutely”, “amazingly”)
  - negation words
  - aggressive language and profanity words: for English, we used the Google Profanity list (https://code.google.com/archive/p/badwordslist/downloads). For Dutch, a public profanity lexicon was consulted (http://scheldwoorden.goedbegin.nl).
  Person alternation is a binary feature indicating whether the combination of a first and second person pronoun occurs in order to capture interpersonal intent.
- **Subjectivity lexicon features:** positive and negative opinion word ratios, as well as the overall post polarity were calculated using existing sentiment lexicons. For Dutch, we made use of the Duoman [71] and Pattern [72] lexicons. For English, we included the Liu and Hu opinion lexicon [73], the MPQA lexicon [74], the General Inquirer Sentiment Lexicon [75], AFINN [76], and MSOL [77]. For both languages, we included the relative frequency of all 68 psychometric categories in the Linguistic Inquiry and Word Count (LIWC) dictionary for English [78] and Dutch [79].
- **Topic model features:** by making use of the Gensim topic modelling library [80], several LDA [81] and LSI [82] topic models with varying granularity (*k* = 20, 50, 100 and 200) were trained on data corresponding to each fine-grained category of a cyberbullying event (e.g. threats, defamations, insults, defenses). The topic models were based on a background corpus (EN: ± 1,200,000 tokens, NL: ± 1,400,000 tokens) scraped with the BootCaT [83] web-corpus toolkit. BootCaT collected ASKfm user profiles using lists of manually determined seed words that are characteristic of the cyberbullying categories.

When applied to the training data, this resulted in 871,296 and 795,072 features for English and Dutch, respectively.

## Results

In this section, we present the results of our experiments to automatically detect cyberbullying signals in an English and Dutch corpus of ASKfm posts. Ten-fold cross-validation was performed in exhaustive grid search over different feature type and hyperparameter combinations (see the Experimental setup section). The *unoptimised word n-gram-based* classifier and *keyword-matching* system serve as baselines for comparison. Precision, Recall and F_1_ performance metrics were calculated on the positive class. We also report Area Under the Receiver Operator Curve (AUROC) scores, a performance metric that is more robust to data imbalance than precision, recall and F score [84].

Table 5 gives us an indication of which feature type combinations score best and hence contribute most to this task. It presents the cross-validation and hold-out scores of a set of feature combinations, which are explained in the feature groups legend (Table 6). A total of 31 feature type combinations, each with 28 different hyperparameter sets have been tested. Table 5 shows the results for the three best scoring systems by included feature types with optimised hyperparameters. The maximum obtained F_1_ score in cross-validation is 64.26% for English and 61.20% for Dutch and shows that the classifier benefits from a variety of feature types. The results on the hold-out test set show that the trained systems generalise well on unseen data, indicating little under- or overfitting. The simple keyword-matching baseline system has the lowest performance for both languages even though it obtains high recall for both languages, especially for English (80.14%), suggesting that profane language characterises many cyberbullying-related posts. Feature group and hyperparameter optimisation provides a considerable performance increase over the unoptimised word *n*-gram baseline system. The top-scoring systems for each language do not differ a lot in performance, except the best system for Dutch, which trades recall for precision when compared to the runner-ups.

**Table 5:** Cross-validated and hold-out scores (%) according to different metrics (F_1_, precision, recall, accuracy and area under the curve) for the English and Dutch three best and worst combined feature type systems.

	Feature combination	Cross-validation scores					Hold-out scores				
		F_1_	P	R	Acc	AUROC	F_1_	P	R	Acc	AUROC
**English**											
Best three	B + C + D + E	**64.26**	73.32	57.19	96.97	78.07	63.69	74.13	55.82	97.21	77.47
	A + B + C	64.24	73.22	57.23	96.96	78.09	**64.32**	74.08	56.83	97.24	77.96
	A + C + E	63.84	73.21	56.59	96.94	77.78	62.94	72.82	55.42	97.14	77.24
Worst three	D	40.48	38.98	42.12	94.10	69.41	39.56	39.56	39.56	94.71	68.39
	A + D + E	38.95	31.47	51.10	92.37	72.76	40.71	33.87	51.00	93.49	73.22
	E	17.35	9.73	79.91	63.72	71.41	15.70	8.72	78.51	63.07	70.44
Baseline	word *n*-gram	58.17	67.55	51.07	96.54	74.93	59.63	69.57	52.17	96.57	75.50
	profanity	17.17	9.61	80.14	63.73	71.53	17.61	9.90	78.51	63.79	71.34
**Dutch**											
Best three	A + B + C + E	**61.20**	56.76	66.40	94.47	81.42	58.13	54.03	62.90	94.58	79.75
	A + B + C + D + E	61.03	71.55	53.20	95.53	75.86	**58.72**	67.40	52.03	95.62	75.21
	A + C + E	60.82	71.66	52.84	95.53	75.68	58.15	67.71	50.96	95.61	74.71
Worst three	D + B	32.90	29.23	37.63	89.91	65.61	30.16	34.72	26.65	92.61	61.73
	D	28.65	19.36	55.10	81.97	69.48	25.13	16.73	50.53	81.99	67.26
	B	24.74	21.24	29.61	88.16	60.94	17.99	23.15	14.71	91.98	55.80
Baseline	word *n*-gram	50.39	67.80	40.09	94.81	69.38	49.54	64.29	40.30	95.09	69.44
	profanity	28.46	19.24	54.66	81.99	69.28	25.13	16.73	50.53	81.99	67.26

**Table 6:** Feature group mapping (Table 5).

A	word *n*-grams
B	subjectivity lexicons
C	character *n*-grams
D	term lists
E	topic models

Table 7 presents the scores of the (hyperparameter-optimised) single feature type systems, to gain insight into the performance of these feature types when used individually. Analysis of the combined and single feature type sets reveals that **word *n*-grams, character *n*-grams**, and **subjectivity lexicons** prove to be strong features for this task. In effect, adding character *n*-grams always improved classification performance for both languages. They are likely to provide robustness to lexical variation in social media text, as compared to word *n*-grams. While subjectivity lexicons appear to be discriminative features, term lists perform badly on their own as well as in combinations for both languages. This shows once again (see the profanity baseline) that cyberbullying detection requires more sophisticated information sources than profanity lists. Topic models seem to do badly for both languages on their own, but in combination with other features, they improve Dutch performance consistently. A possible explanation for their varying performance in both languages would be that the topic models trained on the Dutch background corpus are of better quality than the English ones. In effect, a random selection of background corpus texts reveals that the English scrape contains more noisy data (i.e. low word-count posts and non-English posts) compared to the Dutch scraped corpus.

**Table 7:** Cross-validated and hold-out scores (%) according to different metrics (F_1_, precision, recall, accuracy and area under the ROC curve) for English and Dutch single feature type systems.

	Feature type	Cross-validation scores					hold-out scores				
		F_1_	P	R	Acc	AUROC	F_1_	P	R	Acc	AUROC
**English**											
	word *n*-grams	**60.09**	60.49	59.69	96.22	78.87	**58.35**	57.12	59.64	96.27	78.79
	subjectivity lexicons	56.82	73.32	46.38	96.64	72.77	56.16	72.61	45.78	96.87	72.50
	character *n*-grams	52.69	58.70	47.80	95.91	73.06	53.33	62.37	46.59	96.43	72.65
	term lists	40.48	38.98	42.12	94.10	69.41	39.56	39.56	39.56	94.71	68.39
	topic models	17.35	9.73	79.91	63.72	71.41	15.70	8.72	78.51	63.07	70.44
**Dutch**											
	word *n*-grams	**55.53**	72.64	44.94	95.27	71.88	**54.99**	70.20	45.20	95.57	71.99
	subjectivity lexicons	54.34	54.12	54.56	93.97	75.65	51.82	50.61	53.09	94.09	74.90
	character *n*-grams	51.70	67.58	41.86	94.86	70.22	50.46	65.20	41.15	95.17	69.88
	term lists	28.65	19.36	55.10	81.97	69.48	25.13	16.73	50.53	81.99	67.26
	topic models	24.74	21.24	29.61	88.16	60.94	17.99	23.15	14.71	91.98	55.80

A shallow **qualitative analysis** of the classification output provided insight into some of the classification mistakes.

Table 8 gives an overview of the error rates per cyberbullying category of the best performing and baseline systems. This could give an indication of the types of bullying are hard to detect by the current classifier. All categories are always considered positive for cyberbullying (i.e. the error rate equals the false negative rate), except for *Sexual* and *Insult* which can also be negative (in case of harmless sexual talk and ‘socially acceptable’ insulting language like “hi bitches, in for a movie?” the corresponding category was indicated, but the post itself was not annotated as cyberbullying) and *Not cyberbullying*, which is always negative. Error rates often being lowest for the profanity baseline confirms that it performs particularly well in terms of recall (at the expense of precision, see Table 5). When looking at the best system for both languages, we see that *Defense* is the hardest category to classify. This should not be a surprise as the category comprises defensive posts from bystanders and victims, which contain less aggressive language than cyberbullying attacks and are often shorter in length than the latter. Assertive defensive posts (i.e. a subcategory of *Defense*) which attack the bully are, however, more often correctly classified. There are not sufficient instances of the *Encouragement* class for either language in the hold-out set to be representative. In both languages, threats, curses and incidences of sexual harassment are most easily recognisable, showing (far) lower error rates than the categories *Defamation*, *Defense*, *Encouragements to the harasser*, and *Insult*.

**Table 8:** Error rates (%) per cyberbullying subcategory on hold-out for English and Dutch systems.

	Category	Nr. occurrences in hold-out	Profanity baseline	Word *n*-gram baseline	Best system
**English**					
	Curse	*n* = 109	14.68	30.28	24.77
	Defamation	*n* = 21	23.81	47.62	38.10
	Defense	*n* = 165	22.42	52.12	43.64
	Encouragement	*n* = 1	0.00	100.00	100.00
	Insult	*n* = 345	26.67	41.74	35.94
	Sexual	*n* = 165	63.80	21.47	21,47
	Threat	*n* = 12	8.33	41.67	25.00
	Not cyberbullying	*n* = 10,714	36.94	1.10	0.76
**Dutch**					
	Curse	*n* = 96	39.58	50.00	22.92
	Defamation	*n* = 6	100.00	66.67	33.33
	Defense	*n* = 200	52.50	63.50	46.00
	Encouragement	*n* = 5	40.00	60.00	40.00
	Insult	*n* = 355	43.38	47.89	28.17
	Sexual	*n* = 37	37.84	21.62	27.03
	Threat	*n* = 15	33.33	46.67	20.00
	Not cyberbullying	*n* = 7,295	15.63	1.23	3.07

A qualitative error analysis of the English and Dutch predictions reveals that false positives often contain aggressive language directed at a second person, often denoting personal flaws or containing sexual and profanity words. We see that misclassifications are often short posts containing just a few words and that false negatives often lack explicit verbal signs of cyberbullying (e.g. insulting or profane words) or are ironic (examples 2 and 3). Additionally, we see that cyberbullying posts containing misspellings or grammatical errors and incomplete words are also hard to recognise as such (examples 4 and 5). The Dutch and English corpus are overall similar with respect to qualitative properties of classification errors.

2 *You might want to do some sports* *ahah x*
3 *Look who is there… my thousandth anonymous hater*, *congratulations*!
4 *ivegot 1 word foryou… yknow whatit is*? *→ slut*
5 *One word for you*: *G—A—…*

In short, the experiments show that our classifier clearly outperforms both a keyword-based and word *n*-gram baseline. However, analysis of the classifier output reveals that false negatives often lack explicit clues that cyberbullying is going on, indicating that our system might benefit from irony recognition and integrating world knowledge to capture such implicit realisations of cyberbullying.

Our annotation scheme allowed to indicate different author roles, which provides better insight into the realisation of cyberbullying. Table 9 presents the error rates of our classifier for the different author roles, being harasser, victim, and two types of bystanders. We observe that the error rates are high for *bystander assistant* and *victim*, but there are not sufficient instances in the hold-out set of the former role for either language to be representative. Error rates for the *victim* class of 50.39% and 54% in English and Dutch respectively indicate that the role is hard to recognise by the classifier. A possible explanation for this could be that victim posts in our corpus either expressed powerlessness facing the bully (example 6) or either contained explicit aggressive language as well (example 7).

6 *Your the one going round saying im a cunt and a twat and im ugly. tbh all im doing is sticking up for myself*.
7 *You’re fucked up saying I smell from sweat*, *because unlike some other people I shower every day BITCH*

**Table 9:** Error rates (%) per cyberbullying participant role on hold-out for English and Dutch systems.

	Participant role	Nr. occurrences in hold-out	Profanity baseline	Word *n*-gram baseline	Best system
**English**					
	Harasser	*n* = 328	20.43	48.48	43.60
	Bystander assistant	*n* = 2	50.00	100.00	100.00
	Bystander defender	*n* = 39	7.69	38.46	25.64
	Victim	*n* = 129	27.91	57.36	50.39
	Not cyberbullying	*n* = 10872	37.64	1.24	0.89
**Dutch**					
	Harasser	*n* = 261	47.13	56.70	29.89
	Bystander assistant	*n* = 6	50.00	66.67	50.00
	Bystander defender	*n* = 52	25.00	38.46	23.08
	Victim	*n* = 150	62.00	72.00	54.00
	Not cyberbullying	*n* = 7370	16.01	1.42	3.41

According to the figures, the most straightforward roles in detection are *bystander defender* and *harasser*.

In the light of comparison with state-of-the-art approaches to cyberbullying, we observe that competitive results are obtained with regard to [30–32, 41]. However, the fundamental differences with respect to data collection, sources, and conceptualisations of bullying hardly allow for direct comparison. Table 10 presents the experimental results obtained by [43–45] who, like the current study, approach the task as detecting posts from bullies as well as from victims and bystanders. Given their experimental setup (i.e. task description, data genre and classifier), their work can be considered most similar to ours so their results might function as benchmarks. Also here, a number of crucial differences with the current approach can be observed: Firstly, their corpora were collected using the keywords “bully”, “bullying” and “bullied”, which may bias the dataset towards the positive class and ensures that many explicit lexicalisations are present in the positive class. Second, it is not clear which types of cyberbullying (i.e. explicit and implicit bullying, threats, insults, sexual harassment) are included in the positive class. Furthermore, as can be deduced from Table 10, the datasets are considerably smaller than ours and show a more balanced class distribution (respectively 39% cyberbullying posts in [43] and [44], and 29%/26% in [45]) than the ratio of bullying posts in our corpus (see Table 3: 5% for English, 7% for Dutch). Hence, any comparison should be made with caution due to these differences.

**Table 10:** Overview of the most related cyberbullying detection approaches.

Reference	Classifier	Corpus	Bully rate	F_1_ score
[44]	SVM	1,762 tweets	39%	77%
[43]	wvec+SVM	1,762 tweets	39%	78%
[45]	smSDA+SVM	7,321 tweets	29%	72%
[45]	smSDA+SVM	1,539 MySpace posts	26%	78%

These studies obtain higher scores on similar task but vastly different datasets. Notably, [45] shows a great improvement in classification performance using deep representational learning with a semantic-enhanced marginalized denoising auto-encoder over traditional *n*-gram and topic modelling features.

## Conclusions and future research

The goal of the current research was to investigate the automatic detection of cyberbullying-related posts on social media. Given the information overload on the web, manual monitoring for cyberbullying has become unfeasible. Automatic detection of signals of cyberbullying would enhance moderation and allow to respond quickly when necessary.

Cyberbullying research has often focused on detecting cyberbullying ‘attacks’ and hence overlook other or more implicit forms of cyberbullying and posts written by victims and bystanders. However, these posts could just as well indicate that cyberbullying is going on. The main contribution of this paper is that it presents a system to automatically detect **signals of cyberbullying** on social media, including different types of cyberbullying, covering posts from bullies, victims and bystanders. We evaluated our system on a manually annotated cyberbullying corpus for English and Dutch and hereby demonstrated that our approach can easily be applied to different languages, provided that annotated data for these languages are available.

A set of binary classification experiments were conducted to explore the feasibility of automatic cyberbullying detection on social media. In addition, we sought to determine which information sources contribute most to the task. Two classifiers were trained on an English and Dutch ASKfm corpus and evaluated on a hold-out test of the same genre. Our experiments reveal that the current approach is a promising strategy for detecting signals of cyberbullying on social media automatically. After feature and hyperparameter optimisation of our models, a maximum F_1_ score of 64.32% and 58.72% was obtained for English and Dutch, respectively. The classifiers hereby significantly outperformed a keyword and an (unoptimised) *n*-gram baseline. A qualitative analysis of the results revealed that false positives often include implicit cyberbullying or offenses through irony, the challenge of which will constitute an important area for future work. Error rates on the different author roles in our corpus revealed that especially victims are hard to recognise, as they react differently in our corpus, showing either powerlessness facing the bully or reacting in an assertive and sometimes even aggressive way.

As shown in [45] deep representation learning is a promising avenue for this task. We therefore intent to apply deep learning techniques to improve classifier performance.

Another interesting direction for future work would be the detection of fine-grained cyberbullying categories such as threats, curses and expressions of racism and hate. When applied in a cascaded model, the system could find severe cases of cyberbullying with high precision. This would be particularly interesting for monitoring purposes. Additionally, our dataset allows for detection of participant roles typically involved in cyberbullying. When applied as moderation support on online platforms, such a system enables feedback in function of the recipient (i.e. a bully, victim, or bystander).
